# Non-Markovian effects on protein sequence evolution due to site dependent substitution rates

**DOI:** 10.1186/s12859-016-1135-1

**Published:** 2016-06-24

**Authors:** Francesca Rizzato, Alex Rodriguez, Alessandro Laio

**Affiliations:** International School for Advanced Studies (SISSA), Via Bonomea 265, Trieste, 34136 Italy

**Keywords:** Non-Markovian evolution, Amino acid substitution matrices, Substitution rate variability, Evolutionary distances, Protein sequence evolution

## Abstract

**Background:**

Many models of protein sequence evolution, in particular those based on Point Accepted Mutation (PAM) matrices, assume that its dynamics is Markovian. Nevertheless, it has been observed that evolution seems to proceed differently at different time scales, questioning this assumption. In 2011 Kosiol and Goldman proved that, if evolution is Markovian at the codon level, it can not be Markovian at the amino acid level. However, it remains unclear up to which point the Markov assumption is verified at the codon level.

**Results:**

Here we show how also the among-site variability of substitution rates makes the process of full protein sequence evolution effectively not Markovian even at the codon level. This may be the theoretical explanation behind the well known systematic underestimation of evolutionary distances observed when omitting rate variability. If the substitution rate variability is neglected the average amino acid and codon replacement probabilities are affected by systematic errors and those with the largest mismatches are the substitutions involving more than one nucleotide at a time. On the other hand, the instantaneous substitution matrices estimated from alignments with the Markov assumption tend to overestimate double and triple substitutions, even when learned from alignments at high sequence identity.

**Conclusions:**

These results discourage the use of simple Markov models to describe full protein sequence evolution and encourage to employ, whenever possible, models that account for rate variability by construction (such as hidden Markov models or mixture models) or substitution models of the type of Le and Gascuel (2008) that account for it explicitly.

**Electronic supplementary material:**

The online version of this article (doi:10.1186/s12859-016-1135-1) contains supplementary material, which is available to authorized users.

## Background

Since the publication of the work by Dayhoff and Eck [1] introducing for the first time the concept of PAM matrices, protein sequence evolution has been typically modeled as a time-homogeneous Markov process and each protein site is assumed to be ruled by the same dynamic laws and to evolve independently from the others and from its own past history. This concept is a milestone in the modeling of protein evolution and is, for example, at the basis of several successful approaches for structure prediction. After Dayhoff’s first paper, PAM matrices have been further developed and specialized by using larger datasets [2, 3] and different methods to infer the instantaneous substitution rate matrix [4–6]. However, in more recent years, the availability of larger and larger substitution datasets has started challenging this theoretical framework. For example, Benner et al. [7] and Mitchison and Durbin [8] observed qualitative differences in protein evolution at different sequence divergence, raising concerns on treating the substitution process as Markovian. Even more recently, Kosiol and Goldman [9] proved that, if the substitution process is Markovian at the codon level, it is not Markovian at the amino acid level. With that paper it became evident that substitution matrices on codons [10–12] should be preferred to those on amino acids, but it is still unclear up to which point evolution at the codon level can be considered Markovian. In particular, substitution matrices both on amino acids and on codons tend to present high rates for double and triple instantaneous substitution rates, i.e. substitutions between codons differing by more than one nucleotide or between amino acids whose codons differ all for more the one nucleotide. This phenomenon, according to biochemical wisdom, does not seem realistic and may hint to further violation of the Markov assumption not kept into account even when describing the evolution at the codon level.

Another important result in the description of protein sequence evolution was obtained in 1993 by Yang [13, 14], who proved that the estimations of evolutionary distances and evolutionary trees improve if the variability of substitution rates over sites is accounted for. This rate variability, which is typically modeled by a gamma distribution [13, 14], is due to many effects, including different structural and functional constraints [15] and coevolution inducing a coupling between substitutions at close-by sites [16, 17].

The importance of taking rate variability into account is widely recognized in phylogenetics and many methods have been developed to include it when dealing with large multiple sequence alignments [18–21]. However, these findings are generally neglected when building substitution matrices or applying them to alignments where no further information on the rate distribution is available. One noteworthy exception is due to Le and Gascuel [22], who improved the amino acid replacement matrix by Whelan and Goldman [5] by incorporating the variability of evolutionary rates across sites, but still proposing a model on amino acids rather than on codons.

We here present a model based on a Markovian evolution of the single protein site and describe how the among-site variability of substitution rates, by allowing each site to evolve at a different speed, makes the evolution of full protein sequences effectively non-Markovian. The observed non-Markovian behavior at the full-sequence scale can be seen as the consequence of a reduction in the state space: the full state space, consisting in the twenty amino acids on sites characterized by different rates, is implicitly reduced to the simple set of the amino acids, independently of the specific rate of that site and this gives birth to ensemble average transition probabilities on the reduced state space which are not Markovian. The main consequence is that simple Markov models of protein evolution that neglect rate variability (PAM and PAM-like matrices), no matter if they are empirical or mechanistic and if they are developed at the codon or at the amino acid level, are affected by systematic errors that, for example, may lead to underestimating the evolutionary times. We will also show that one of the effects of treating protein evolution as a Markov process is a general overestimation of instantaneous double and triple substitutions, which might explain the corresponding high values found in the most common instantaneous substitution matrices such as the Jones-Taylor-Thornton (JTT) [3], the Whelan and Goldman (WAG) [5] and the Empirical Codon Model (ECM) [10].

## Methods

### Markov models of protein sequence evolution

We first model protein sequence evolution as a homogeneous continuous-time Markov process defined by an *N*×*N* instantaneous substitution matrix, *Q*, where *N* is the number of possible states [23]. When protein evolution is modeled on the amino acids, the possible states are the 20 amino acids and *N*^*AA*^=20, while when the framework of codons is chosen, the possible states are the 61 codons coding for amino acids and *N*^*c*^=61. From now on the superscript ^*c*^ (resp. ^*AA*^) will be reserved to codon related (resp. amino acid related) quantities.

Each off-diagonal entry of *Q*, *Q*_*i,j*≠*i*_, represents the instantaneous substitution rate from state *i* to state *j* and is assumed to be constant in time and over sites. The diagonal entries are defined as minus the sum of all the other entries in that row, $Q_{ii}=-\sum _{j\neq i}Q_{ij}$ and account for the instantaneous probability of escaping from each state. *Q* is normalized so that $\sum _{i}\sum _{j\neq i}\left (\pi _{i}Q_{ij}\right)=1$, where *π*_*i*_ is the equilibrium probability of state *i*, defined by the set of conditions $\sum _{i}\pi _{i}Q_{ij}=0$. Because of this normalization, the time is measured in units of expected substitutions per site. For example, *t*=0.01 corresponds to a typical rate of substitution of 1 *%*, constant along the protein chain.

To analyze the dynamics in the framework of codons we consider the M0 model introduced by Yang [24]:

1 $$\begin{array}{@{}rcl@{}} Q_{i,j\neq i}^{c} & \propto & \left\{ \begin{array}{cc} 0 & i\:\text{or}\: j\:\text{stop codons}\\ 0 & i\rightarrow j\:>1\:\text{nucl. subst.}\\ {\pi_{j}^{c}} & i\rightarrow j\ \text{syn. transv.}\\ {\pi_{j}^{c}}\kappa & i\rightarrow j\ \text{syn. transit.}\\ {\pi_{j}^{c}}\omega & i\rightarrow j\:\text{nonsyn. transv.}\\ {\pi_{j}^{c}}\kappa\omega & i\rightarrow j\:\text{nonsyn. transit}. \end{array}\right. \end{array} $$

where ${\pi _{j}^{c}}$ is the equilibrium probability for codon *j*, *κ* is the transition/transversion rate ratio and *ω* is the nonsynonymous/synonymous rate ratio. The parameters are set to their typical values for protein-coding DNA: *ω*=0.2,*κ*=2.5 and the codon distribution ${\pi _{i}^{c}}$ is chosen as in Kosiol and Goldman [9]. For the sake of completeness, the substitution dynamics is here modeled also in the amino acid framework, using the WAG matrix [5] as *Q*^*AA*^.

The transition probability from state *i* to state *j* in a time interval of *t* is given by:

2 $$ P_{ij}(t)=\left[e^{tQ}\right]_{ij}  $$

The matrix *P*(*t*), defined from now on as the *transition probability matrix at time t*, describes by construction a Markovian dynamics.

### Ensemble average transition probabilities

We now consider the effect on protein sequence evolution of a site-dependent substitution rate. Consistently with what proposed by Yang [13], we assume that the rate of substitution, *r*, is distributed according to a *γ*-shaped probability density function with average value 〈*r*〉=1:

3 $$ \rho(r)=k\cdot exp\left(-\alpha\cdot r\right)r^{\left(\alpha-1\right)}  $$

where *α* is the shape parameter and *k* is a normalization factor such that $\int _{0}^{\infty }\rho (r)dr=1$. In the rest of the paper, both in the codon and in the amino acid model, we are going to use *α*=0.286. This value is consistent with those observed in several protein families (see Table 1 of ref. [25]), but the specific choice of *α* is here irrelevant, being the scope of this work to provide a demonstration of the consequences of a plausible rate distribution on protein sequence evolution. The results for other values of *α* in the range [0.2:1] are reported in the Additional files.

The transition probability from state *i* to state *j* in a time interval of *t* for a site characterized by rate *r* is given by:

4 $$ P_{ij}\left(r,t\right)=\left[e^{r\cdot t\cdot Q}\right]_{ij}  $$

When no information is available on the specific rate of each site, which is the typical premise when using substitution matrices, we can score an alignment only by comparing it to average transition probabilities. So we are interested in estimating the average probability for a site being in state *i* at time zero to be in state *j* at time *t*, considering that the rate distribution is given by Eq. 3 and that each site evolves according to the Markovian dynamics described by Eq. 4:

5 $$\begin{array}{@{}rcl@{}} \widetilde{P}_{ij}^{c}(t)&=&\int_{0}^{\infty}P_{ij}^{c}\left(r,t\right)\rho(r)dr \\ &=&\left[\int_{0}^{\infty}e^{r\cdot t\cdot Q^{c}}\rho(r)dr\right]_{ij} \end{array} $$

We will call $\widetilde {P}(t)$ the *ensemble average transition probability matrix at time t*. Here the term *ensemble average* should be intended as an average over the ensemble of sites subject to the distribution of the substitution rate described by Eq. 3. We want to highlight that the definition of Eq. 5 implicitly entail that each site is characterized by a substitution rate that remains constant over time. This is, of course, an approximation, because during evolution the propensity for a site to accept mutations may change [26], but, for short evolutionary times and in the range of sequence identity considered in this study (∼80 *%*), this approximation should hold. In fact, this is the same approximation implicitly used in the vast majority of phylogenetic algorithms for tree reconstruction, where each protein site is assumed to maintain the same rate along the branches of the full tree.

### Non-Markovian behavior of ensemble average transition probabilities

According to Eq. 5, the ensemble average transition probability matrix is a combination of many Markovian transition probabilities and, in general, combinations of non-identical Markov processes are not Markovian. In other words, even if here the single-site dynamics is assumed to be Markovian, when the full protein sequence evolution is approximated by neglecting site specificity as done in general substitution matrices, the state space is implicitly reduced and only some special reductions, with respect to which that process is “lumpable” [27], still give rise to Markovian dynamics. With this in mind, the non-Markovian behavior of the full protein sequence dynamics can be simply proved either by checking that $\widetilde {P}(t)\neq \left [\widetilde {P}(\tau)\right ]^{t/\tau } $, namely that $\widetilde {P}$ violates the Chapman-Kolmogorov equation, or by exploiting the properties of lumpable Markov processes. A numerical example of the violation to the Chapman-Kolmogorov equation can be found in Appendix A, while a demonstration of non-Markovianity based on the properties of lumpability can be found in Appendix B.

In the next paragraph, we are going to quantify the entity of the violation of the Markov assumption.

## Results

In order to understand qualitatively the effects of the variation of the rate over sites, let us first consider a simplified world with only three codons, A, B and C, characterized all by the same frequency $\pi _{A}= \pi _{B}= \pi _{C}= \frac {1}{3}$. We assume that the instantaneous substitution matrix for this model is:

$$Q=\left(\begin{array}{ccc} -1 & 0.9 & 0.1\\ 0.9 & -1.1 & 0.2\\ 0.1 & 0.2 & -0.3 \end{array}\right) $$

If the rate of substitution is constant over sites, the transition probability matrix at time *t* is

6 $$ P(t)=e^{tQ}  $$

and describes a Markovian dynamics. On the other hand, we can imagine a sequence where, still keeping the same average rate, half of the sites has a reduced substitution rate of 0.5 and the other half has a faster substitution rate of 1.5. For this second system the ensemble average transition probability matrix at time *t* will be

7 $$ \widetilde{P}(t)=\frac{e^{0.5\cdot tQ}+e^{1.5\cdot tQ}}{2}   $$

**Fig. 1:**
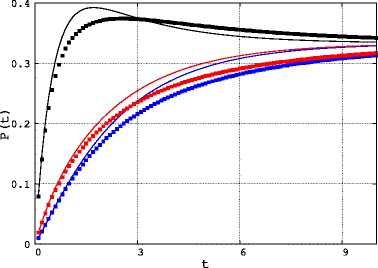
Transition probabilities in a simplified world. Comparison between the transition probabilities in a sequence with constant substitution rate over sites and in a sequence with two equiprobable classes of rates for a simplified system described in Results. *Black*: *P*_*AB*_(*t*) (*solid line*) and $\tilde {P}_{AB}(t)$ (*points*); *Blue*: *P*_*AC*_(*t*) (*solid line*) and $\tilde {P}_{AC}(t)$ (*points*); *Red*: *P*_*BC*_(*t*) (*solid line*) and $\tilde {P}_{BC}(t)$ (*points*)

It may be of interest to monitor the value of these two sets of transition probabilities as functions of time and compare them. Being the three codons equiprobable, *Q*, *P* and $\widetilde {P}$ are symmetric and so we can limit ourselves to check only 3 different transition probabilities: *P*_*AB*_=*P*_*BA*_,*P*_*AC*_=*P*_*CA*_ and *P*_*BC*_=*P*_*CB*_. In Fig. 1 we compare the time evolution of these three quantities (respectively in black, blue and red) in the two systems (in solid line for Eq. 6 and points for Eq. 7): clearly $P(t)\neq \widetilde {P}(t)$. So, it is evident that the variation of the rate induces a change in the average transition probabilities even if both *Q* and the average substitution rate do not change. It is also easy to verify that the dynamics described by Eq. 7 is not Markovian (see Methods).

### Non-Markovian behavior in the framework of codons

A similar effect can be observed in the models describing protein sequence evolution both at the codon level and at the amino acid level and it may be interesting to quantify the violation of the Markov assumption in presence of a plausible rate variation.

**Fig. 2:**
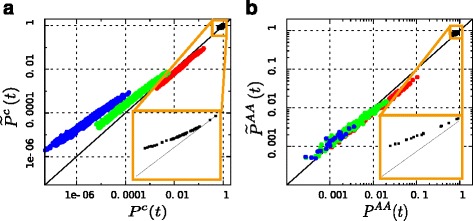
Comparison between Markovian and non-Markovian substitution probabilities in the framework of codons and of amino acids. **a** Points: entry-by-entry comparison of *P* ^*c*^(*t*) and $\widetilde {P}^{c}(t)$ in log-log scale, with *t*=0.235. Each point corresponds to a pair *i,j* of codons and its *x*-value is given by $P^{c}_{ij}(t)$, while its *y*-value is $\widetilde {P}_{ij}^{c}(t)$. The black squares (zoomed in the yellow inset) are the entries with *i*=*j*, while red, green and blue points are respectively the entries where codon *i* and codon *j* differ by one, two or three nucleotides. Solid line: line *y*=*x*. **b** Points: entry-by-entry comparison of *P* ^*AA*^(*t*) and $\widetilde {P}^{AA}(t)$ in log-log scale, with *t*=0.23. Coordinates and lines have the same meaning as in panel (**a**) and colors are such that the entries where *i*=*j* are black (zoomed in the yellow inset), while red, green and blue identify the entries with *i*≠*j* where the most similar pair of codons coding for amino acids *i* and *j* differ respectively by one, two or three nucleotides

Concerning codons, we model the instantaneous substitution matrix as in Eq. 1 and the rate distribution by Eq. 3 (see Methods). Since now the rate distribution is continuous, the sum in Eq. 7 is replaced by an integral and gives Eq. 5. To quantify the variation of the ensemble average transition probability matrix with respect to the Markovian transition probability matrix, we compare *P*^*c*^(*t*) to $\widetilde {P}^{c}(t)$ at time *t*=0.235, which corresponds, for *P*^*c*^, to the 80 % of sequence identity. In Fig. 2a we show the entry-by-entry comparison between them in log-log scale: each point corresponds to a pair *i,j* of codons and its *x*-value is given by the Markovian evolution $P^{c}_{ij}(t)$, while its *y*-value is its non-Markovian counterpart $\widetilde {P}_{ij}^{c}(t)$. If the two dynamics gave the same results, the points would lie on the line *y*=*x*, but this is not the case. In particular, one can see four separate subsets: the black squares (zoomed in the inset) are the entries corresponding to *j*=*i* (the diagonal terms in the matrix), while the red, green and blue points correspond to *j*≠*i* (the off-diagonal ones), where *i* and *j* differ respectively by one, two or three nucleotides. It is evident that, with respect to the Markovian dynamics, $\widetilde {P}^{c}(t)$ gives rise to higher entries for *j*=*i* enhances double and triple substitutions and discourages single ones.

A first consequence is that the expected sequence identity between two sequences separated by an evolutionary time *t* is much lower for the Markovian dynamics than for the non-Markovian one. This happens because, even if the average rate of substitutions is the same, in the non-Markovian case it is much more likely that substitutions cumulate on the few sites with rate larger than 1. In this way, a much larger fraction of substitutions takes place on a site that has already mutated, without further modifying the global sequence identity. The Markovian assumption produces therefore a systematic underestimation of evolutionary times. This result may be considered the theoretical explanation of the observation by Yang et al. [14] that, when taking substitution rate variability into account, one gets larger estimates of branch lengths in phylogenetic trees. The difference of sequence identity between two sequences separated by a given evolutionary time in the two processes can be found in Additional file 3 (Figure S2 (a)). In particular, at the time *t*=0.235 the non-Markovian dynamics presents the 85.7 % of sequence identity, while the Markovian one only the 80 %.

It is, then, more appropriate to compare the two processes at fixed sequence identity: in Additional file 3 (Figure S2 (b)), one can find the same comparison of Fig. 2a with the time $\tilde {t}$ of the Non-Markovian process chosen to produce a sequence identity of the 80 %, which gives $\tilde {t}=0.4$. Even if this choice balances the entries corresponding to *i*=*j*, the non-Markovian dynamics still enhances double and triple substitutions with respect to its Markovian analogue. For example, at sequence identity of 80 %, the estimated probability of finding a substitution from codon ATC to codon TGG (3 different nucleotide, so one of the blue points in Fig. 2a–c) is 5.21·10^−8^ when the Markovian approximation is adopted, while is more than one hundred times bigger if the rate is *γ*-distributed. In Table 1 one can find some other examples of how transition probabilities change in the two frameworks.

**Table 1:** Examples of the variation of the transition probabilities at the sequence identity of 80 % between Markovian (*P*) and non-Markovian ($\widetilde {P}$) dynamics

Initial state	Final state	*P*(*t*)	$\widetilde {P}(\tilde {t})$	$P(t)/\widetilde {P}(\tilde {t})$
ATC	TGG	5.21·10^−8^	8.23·10^−6^	0.006
TTC	ATG	3.39·10^−5^	3.09·10^−4^	0.110
GTC	GTT	0.1507	0.0951	1.58
Ile	Val	0.104	0.076	1.4
Arg	Lys	0.064	0.049	1.3
Gly	Ile	0.0006	0.0022	0.3

The first three rows involve substitutions in the framework of codons, while the last three are in the framework of amino acids

In the Additional files we provide the ensemble average transition probability matrices for codons estimated by Eq. 5 (Additional file 1) and the Markovian counterpart described by Eq. 2 (Additional file 2) at sequence identities ranging from 95 to 50 %.

### Non-Markovian behavior in the framework of amino acids

The same calculations can be also performed in the framework of amino acids: Fig. 2d shows the entry-by-entry comparison between the Markovian dynamics described by *P*^*AA*^ and its non-Markovian analogue $\widetilde {P}^{AA}(t)$ in log-log scale, where *t*=0.23, corresponding to the 80 % of sequence identity in the Markovian dynamics. Here red, green and blue points identify the transition probabilities between pairs of amino acids whose most similar pair of codons (i.e. the pair of codons with maximal number of identical letters) differ respectively by one, two or three nucleotides. Not surprisingly, the subsets are less separated then in the framework of codons. In fact many amino acid substitutions are a combination of single, double and triple nucleotide substitutions. Anyway it can be observed that the substitutions between amino acids where at least two nucleotides must change are more frequent in the non-Markovian dynamics than in the Markovian one, as already observed for codons. The comparison of the sequence identity generated by *P*^*AA*^ and $\widetilde {P}^{AA}$ and the entry-by-entry comparison of *P*^*AA*^(*t*) and $\widetilde {P}^{AA}\left (\tilde {t}\right)$ at the 80 % of sequence identity can be found in Additional file 3 (Figure S2 (c) and (d)). For example, at sequence identity of 80 %, the estimated probability to find a substitution from glycine to isoleucine (where at least two substitutions are needed) is approximately one third for the Markovian model with respect to the non-Markovian one. In Table 1 further examples of differences in the transition probabilities between the Markovian and the non-Markovian dynamics can be found.

### Impact on the estimation of *Q* of the Non-Markovian behavior due to the rate variability

We now show that treating full protein sequence evolution as Markovian, neglecting substitution rate variability, determines also a wrong estimation of *Q*, the instantaneous substitution matrix. In particular, we will see that, when learning *Q*^*c*^ from pairwise alignments, substitution rates between codons differing by more than one nucleotide are systematically magnified. This is somehow intuitive: rate variability allows substitutions to accumulate on the few sites with high substitution rate and so, when learning substitution frequencies from alignments, we find a larger number of double and triple substitutions than expected if the rates were constant. Then, when inferring *Q*^*c*^ from these data without taking rate variability into account, the only way to encompass the extra number of double substitutions is to enhance instantaneous double and triple transition probabilities. For simplicity we are going to show this for a particular case, where *Q*^*c*^ is estimated from alignments all at the same sequence identity, but the reasoning can be generalized for alignments at various sequence identity and for multiple sequence alignments.

To evaluate the order of magnitude of this overestimation of instantaneous double and triple substitutions, we recover a measure of $Q^{c},\widetilde {Q}^{c}(t)$, from the ensemble average transition probability matrix at time $\tilde {t}=0.4,\widetilde {P}^{c}\left (\tilde {t}=0.4\right)$. If, when estimating $\widetilde {Q}^{c}\left (\tilde {t}\right)$, we are considering the process as Markovian, for a sequence identity of 80 % we would infer the evolutionary time being not $\tilde {t}=0.4$ but rather *t*=0.235 (see previous calculations and Fig. 2b). So we can calculate $\widetilde {Q}^{c}\left (\tilde {t}\right)$ by inverting Eq. 2:

8 $$ \widetilde{Q}^{c}\left(\tilde{t}\right)=\frac{log\left(\widetilde{P}^{c}\left(\tilde{t}\right)\right)}{t}  $$

with $\tilde {t}=0.4$ and *t*=0.235.

**Fig. 3:**
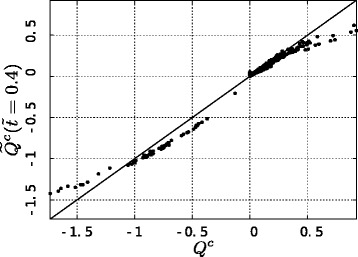
Impact of the Markovian assumption on the estimation of *Q*. *Points*: entry-by-entry comparison between *Q* ^*c*^ and $\widetilde {Q}^{c}(\tilde {t}=0.4)$ estimated by Eq. 8. *Solid line*: *y*=*x*

Figure 3 shows the entry-by-entry comparison between the original *Q*^*c*^ and $\widetilde {Q}^{c}\left (\tilde {t}\right)$. The two matrices clearly do not correspond, as the points do not lie on the line *y*=*x*, so the estimate of *Q*^*c*^ from alignments when neglecting rate variability is affected by systematic errors. In particular, we can calculate the fraction of instantaneous double substitutions in the original $Q^{c},f_{2}^{true}$, and in the estimated $\widetilde {Q}^{c},f_{2}^{est}$, by:

9 $$\begin{array}{@{}rcl@{}} f_{2}^{true} & = & \frac{\sum_{i,j|\:2\neq nucl}\left[\pi_{i}\cdot Q_{ij}^{c}\right]}{\sum_{i,j\neq i}\left[\pi_{i}\cdot Q_{ij}^{c}\right]} \end{array} $$

10 $$\begin{array}{@{}rcl@{}} f_{2}^{est} & = & \frac{\sum_{i,j|\:2\neq nucl}\left[\pi_{i}\cdot\widetilde{Q}_{ij}^{c}\left(\tilde{t}=0.4\right)\right]}{\sum_{i,j\neq i}\left[\pi_{i}\cdot\widetilde{Q}_{ij}^{c}\left(\tilde{t}=0.4\right)\right]} \end{array} $$

where *π*_*i*_ is the equilibrium probability of codon *i* and the sum at the numerator is the restricted sum over the entries involving a pair of codons *i,j* differing by two nucleotides. The fractions of triple substitutions for the original *Q*^*c*^,*f*_3_, and for the estimated $\widetilde {Q}^{c},f_{3}^{est}$, are computed in a similar way.

In the original instantaneous rate matrix *Q*^*c*^ (Eq. 1) double and triple substitutions are not allowed, so $f_{2}^{true}=f_{3}^{true}=0$ by construction, while, in the estimated matrix $\widetilde {Q}^{c}\left (\tilde {t}\right)$, we get $f_{2}^{est}=0.153$ and $f_{3}^{est}=0.017$. So, the sum of the fractions of instantaneous double and triple substitution estimated from alignments at the 80 % of sequence identity would make up the 17 % of all the instantaneous substitutions, while in the original Markovian model they are the 0 %.

This result might cast some light on the anomalous high entries for double and triple substitutions in the *Q* matrix of many models: the sum of the fractions of instantaneous double and triple substitutions is 25 % in the ECM, 22 % in the WAG and 14 % in the JTT matrix (for the last two, double substitutions are defined as the substitutions between amino acids whose most similar pair of codons differ by two nucleotides). Considering that mutations take place by chance, one would rarely expect double or triple substitutions to happen in an infinitesimal time on the same codon, which is also the underlying hypothesis in the definition of the mechanistic *Q*^*c*^ of Eq. 1. For example if single mutations take place with probability *p* in a small time *dt* and the sites evolve independently, two neighbor mutations in the same time interval should happen at the much smaller probability of *p*^2^.

A possible explanation of the high value of double and triple substitution rates in standard substitution matrices is that the Markov assumption may have induced a fictitious increase for double and triple substitutions. A full proof of this idea would require recalculating *Q* from the same alignments used to build each matrix by including the rate variability. However, this explanation is consistent with two previous results: when the WAG matrix was re-examined by Le and Gascuel by including the *γ* correction, they found smaller values for the triple substitutions [22] and De Maio et al. [28] observed that accounting for rate variability by hidden Markov Models reduces the estimate of instantaneous multiple substitutions in the ECM matrix.

## Discussion and conclusions

We have discussed the effects of the among-site variability of substitution rates in the process of protein sequence evolution. The relative difference of the rates mixes Markov processes with different speed, which makes the process of full sequence evolution effectively not Markovian. The first consequence of the violation of the Markov assumption is a systematic underestimation of evolutionary distances. We have quantified the violation of the Markov assumption for two realistic models (respectively on codons and amino acids), demonstrating that neglecting the rate variability may cause two orders of magnitude of difference in the relative probability for triple substitutions and one order of magnitude for double substitutions. We have also shown that this approach modifies in a radical way the estimate of Q itself by especially magnifying double and triple substitutions, which might explain the correspondent high transition probabilities in the main instantaneous substitution matrices (e.g. JTT, WAG, ECM).

Statistical inference of phylogenies under Markov models including *γ*-distributed rate variation [13, 14] as well as CAT models [20] can effectively deal with this problem and mixture models [18, 19, 29], that allow not only site-dependent substitution rates but also site-dependent substitution matrices, can go even beyond. However, the substitution matrices for codons and amino acids are most of the times derived without taking into account the among-site rate variability and these matrices enter necessarily even in the construction of the seed multiple sequence alignment at the basis of any hidden Markov Model. According to our findings, Markovian models for protein evolution based on most of the available substitution matrices are affected by errors that get worse when inferring information far from the learning set. This is valid both for models at the codon level and at the amino acid level, for which Kosiol and Goldman [9] have already showed that a further source of memory is present.

The results shown here are robust with respect to the specific choice of the rate matrix and rate distribution: as can be guessed by the first simple example in Results, any non-trivial rate distribution combined in Eq. 5 with whatever *Q* gives rise to ensemble average transition probabilities $\widetilde {P}$ which differ from the simple *P*=*e*^*tQ*^. The results presented in Fig. 2 should then be intended as a “proof-of-principle” that variable substitution rates cause a non-Markovian full protein sequence evolution and as a plausible estimate of the entity of the systematic errors arising when using standard substitution models in a naive way.

Even if further and more specific analysis would be necessary to quantify the impact of the effect described in this work on specific applications, the present results seem to discourage the use of simple Markovian models that neglect among-site rate variability for both amino acid and codon sequence alignments, especially when the substitution matrices are learned on alignments in a range of sequence identity very different from the test set. On the other hand, they encourage the use of models that account for among-site rate variability, for example mechanistic codon models with the *γ* correction, Hidden Markov Models [28, 30, 31], CAT models [20] or other mixture models [18, 19, 29] that allow it by construction, or substitution models of the type of Le and Gascuel (LG) [22] that account for it explicitly. In particular, we highlight the necessity of developing a codon analogue of the LG matrix, in order to get rid at the same time of both the identified factors leading to a non-Markovian behavior of full protein sequence evolution: the degeneration of the genetic code and the rate variability.

## Appendix A: Numerical proof of the violation of the Markov assumption

In this section we provide a numerical example that proves the violation of the Markov assumption in the evolution ruled by $\widetilde {P}$. For all Markov processes described by a transition probability *P* the following property holds for any pair of times *t*_0_ and *t*:

11 $$ P(t)=\left[P\left(t_{0}\right)\right]^{t/t_{0}}  $$

This equation, generally known as the Chapman-Kolmogorov equation [32], can then be employed as a test of the Markov assumption.

In Additional file 3 (Figure S1) we compare entry by entry in log-log scale $\widetilde {P}^{c}(t)$ with $\left [\widetilde {P}^{c}(t_{0})\right ]^{t/t_{0}}$ for *t*=0.235 and *t*_0_=0.01. Coordinates, colors and lines have the same meaning as in Fig. 2a in section Results. It is evident that the points do not lie on the diagonal so $\widetilde {P}^{c}(t)\neq \left [\widetilde {P}^{c}(t_{0})\right ]^{t/t_{0}}$. Moreover one can notice that $\left [\widetilde {P}^{c}(t_{0})\right ]^{t/t_{0}}$ is much more similar to *P*^*c*^(*t*) than to $\widetilde {P}^{c}(t)$. Indeed $\left [\widetilde {P}^{c}(t_{0})\right ]^{t/t_{0}}=e^{t\cdot \left [ \log \left (\widetilde {P}^{c}(t_{0})\right)/t_{0}\right ]}$ and, being *t*_0_ very small, $\log \left (\widetilde {P}^{c}(t_{0})\right)/t_{0}\simeq Q^{c}$. So, for this choice of *t* and $t_{0},\left [\widetilde {P}^{c}(t_{0})\right ]^{t/t_{0}}$ mimics a Markovian dynamics for a *Q*^*c*^ slightly different from Eq. 1.

## Appendix B: Proof of the violation of the Markov assumption by the properties of lumpable processes

Given a set of states *s*={*s*_1_,*s*_2_,…*s*_*N*_} and a partition on it *A*={*A*_1_,*A*_2_,…*A*_*r*_}, a necessary and sufficient condition for a Markov chain on *s* to be lumpable with respect to *A* is that, for every pair of sets *A*_*i*_ and *A*_*j*_, the sum $\sum _{s_{l}\in Aj} p_{s_{k},s_{l}}$ of the transition probabilities from state *s*_*k*_ to states *s*_*l*_∈*A*_*j*_ has the same value for every *s*_*k*_∈*A*_*i*_ [27].

We exploit this property to prove the non-Markovian behavior of sequence evolution in presence of rate heterogeneity. Here the full state space is given by all the possible pairs {*r,c*} with *r* a real number in [ 0:*∞*] corresponding to a rate value and *c* one of the 64 codons. We consider the following transition probability from state *s*_1_ to state *s*_2_: *p*_{*r*1,*c*1},{*r*2,*c*2}_=*δ*(*r*_1_,*r*_2_)·[*e*^*r*1·*Δt*·*Q*^]_*c*1,*c*2_. where *Δt* is an arbitrarily small time. We partition the state space into a 64-dimensional reduced space given by the set of possible codons: *A*={*c*_1_,*c*_2_,…*c*_64_}, thus each set in *A* contains all the states characterized by a same codon and different rates. The dynamics described by *p*_{*r*1,*c*1},{*r*2,*c*2}_ is lumpable with respect to *A* only if $\int p_{\left \lbrace r,c1 \right \rbrace,\left \lbrace r2,c2 \right \rbrace } dr_{2}=\int p_{\left \lbrace s,c1 \right \rbrace,\left \lbrace r2,c2 \right \rbrace } dr_{2}$ for all the possible *r* and *s*. But the first term gives $ \int \delta (r,r_{2})\cdot \left [e^{rtQ}\right ]_{c1,c2} dr_{2}=\left [e^{rtQ}\right ]_{c1,c2}$ while the second term gives [*e*^*stQ*^]_*c*1,*c*2_ which are equal only if *r*=*s*. So the dynamics of the reduced process, in presence of rate variability, is not Markovian.

## Abbreviations

CAT, mixture model that classifies sites into CATegories. [20]; ECM, empirical codon model [10]; JTT, Jones Taylor Thornton [3]; LG, Le Gascuel [22]; PAM, point accepted mutation [2]; WAG, Whelan And Goldman [5]

## Additional files

Additional file 1 P_NonMarkovian.txt. Ensemble average transition probability matrices for codons according to Eq. 5, where the rate is *γ*-distributed as in Eq. 3, for various *t*. The file is structured in 12 columns: the first two contain *i* and *j*, the other ten $\widetilde {P}^{c}(t)_{ij}$ respectively for *t*=0.06,0.14,0.25,0.4,0.6,0.9,1.3,1.9,2.9,4.3 corresponding to the sequence identities of 95 *%*,90 *%*...55 *%*,50 *%*. *i* and *j* identify codons by alphabetical order: 1=“AAA”, 2=“AAC”... 64=“TTT”. (TXT 550 kb)

Additional file 2 P_Markovian.txt. Probability matrices for codons according to Eq. 2 for various *t*. The file is structured in 12 columns: the first two contain *i* and *j*, the other ten $P^{c}_{ij}(t)$ respectively for *t*=0.05,0.11,0.17,0.235,0.31,0.39,0.48,0.58,0.7,0.84 corresponding to the sequence identities of 95 *%*,90 *%*...55 *%*,50 *%*. *i* and *j* identify codons by alphabetical order: 1=“AAA”, 2=“AAC”... 64=“TTT”. (TXT 550 kb)

Additional file 3 Suppl_Figures.pdf. Contains three supplementary figures. Figure S1 (for Appendix), with the test of the Chapman-Kolmogorov equation. Figure S2, with the comparison of the sequence identity as functions of time and of the transition probabilities at the same sequence identity between the Markovian and the non-Markovian dynamics, both for codons and for amino acids. Figure S3 is a panel containing the entry-by-entry comparison of *P* ^*c*^(*t*) and $\widetilde {P}^{c}(\tilde {t})$ calculated at the same sequence identity for different choice of the shape parameter *α* in the *γ* distribution. (PDF 143 kb)
