# Supplementary material for: Non-Markovian effects on protein sequence evolution due to site dependent substitution rates
Source: BMC Bioinformatics. 2016 Jun 24;17:258. doi: 10.1186/s12859-016-1135-1 (PMC4921000; doi:10.1186/s12859-016-1135-1)
Supplement: Additional file 3 — Suppl_Figures.pdf. Contains three supplementary figures. Figure S1 (for Appendix), with the test of the Chapman-Kolmogorov equation. Figure S2, with the comparison of the sequence identity as functions of time and of the transition probabilities at the same sequence identity between the Markovian and the non-Markovian dynamics, both for codons and for amino acids. Figure S3 is a panel containing the entry-by-entry comparison of P c(t) and $\widetilde {P}^{c}(\tilde {t})$P~c(t~) calculated at the same sequence identity for different choice of the shape parameter α in the γ distribution. (PDF 143 kb) [file 12859_2016_1135_MOESM3_ESM.pdf]

## Figure S1 (supplementary information).

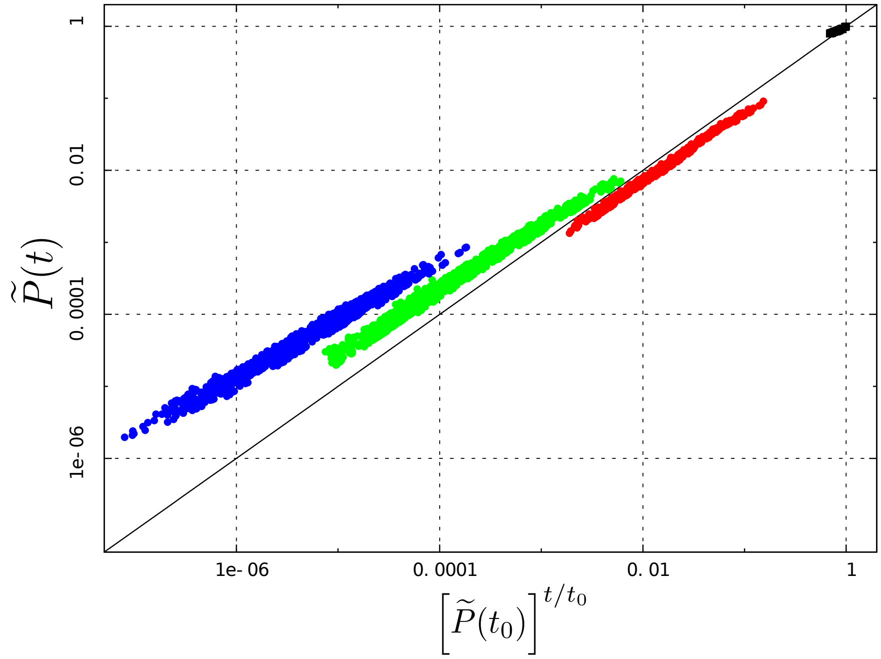


Fig. 1: **Test of the Chapman-Kolmogorov equation.** Points: entry-by-entry comparison of $\left[\widetilde{P}^c\left(t_0\right)\right]^{t/t_0}$ and $\widetilde{P}^c\left(t\right)$ in log-log scale, with $t_0 = 0.01$ and $t = 0.235$. The black squares are the entries with $i = j$, while red, green and blue points are respectively the entries where codon $i$ and codon $j$ differ by one, two or three nucleotides. Solid line: line $y = x$. Points do not lie on the diagonal so the dynamics ruled by $\widetilde{P}$ can not be Markovian.

## Figure S2 (supplementary information).

We can define the average sequence identity between two sequences separated by an evolutionary time $t$ for the Markovian ($I^c_M(t)$) and the non-Markovian ($I^c_{NM}(t)$) dynamics as:

$$I^c_M(t) = \sum_i \pi^c_i \cdot [P^c(t)]_{ii} \quad (1)$$

$$I^c_{NM}(t) = \sum_i \pi^c_i \cdot \left[\widetilde{P}^c(t)\right]_{ii} \quad (2)$$

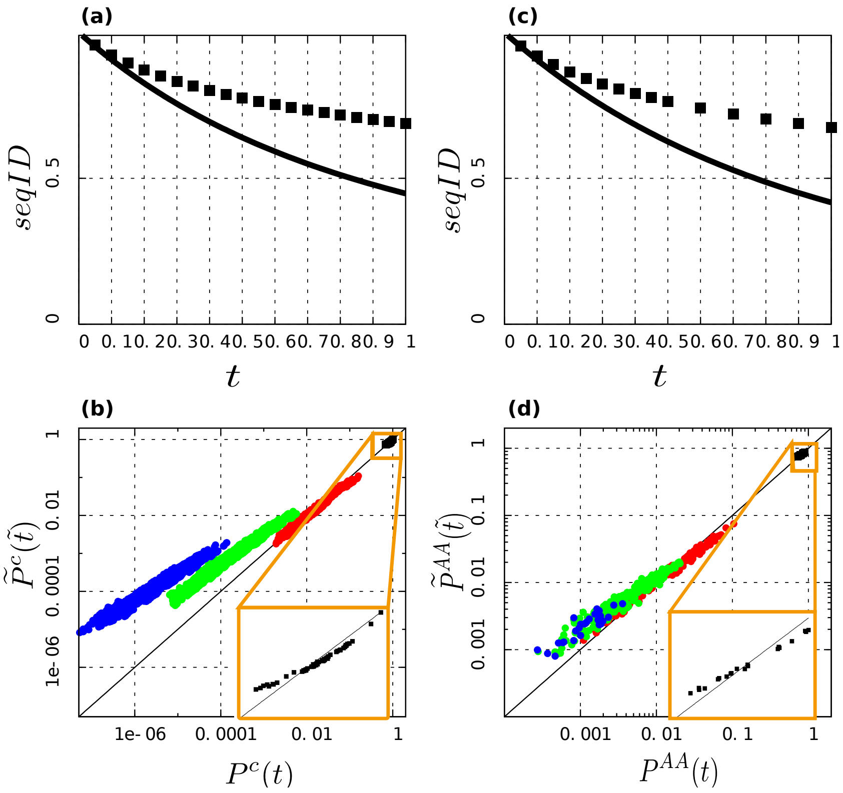


Fig. 2: (a) Comparison of the sequence identity as a function of $t$ for the Markovian ($I^c_M(t)$ in solid line) and the non-Markovian ($I^c_{NM}(t)$ with points) dynamics in the framework of codons. (b) Entry-by-entry comparison of $P^c(t)$ and $\widetilde{P}^c(\tilde{t})$ in log-log scale, with $t = 0.235$ and $\tilde{t} = 0.4$, so that $I^c_M(t) = I^c_{NM}(\tilde{t}) = 0.8$. Each point corresponds to a pair $i, j$ of codons and its $x$-value is given by $P^c_{ij}(t)$, while its $y$-value is $\widetilde{P}^c_{ij}(t)$. The black squares (zoomed in the yellow inset) are the entries with $i = j$, while red, green and blue points are respectively the entries where codon $i$ and codon $j$ differ by one, two or three nucleotides. Solid line: line $y = x$. (c) Comparison of the sequence identity as a function of $t$ for the Markovian (in solid line) and the non-Markovian (with points) dynamics in the framework of amino acids. (d) Points: entry-by-entry comparison of $P^{AA}(t)$ and $\widetilde{P}^{AA}(\tilde{t})$ in log-log scale, with $t = 0.23$ and $\tilde{t} = 0.38$, so that $I^{AA}_M(t) = I^{AA}_{NM}(\tilde{t}) = 0.8$. Coordinates and lines have the same meaning as in panel (a) and colors are such that the entries where $i = j$ are black (zoomed in the yellow inset), while red, green and blue identify the entries with $i \neq j$ where the most similar pair of codons coding for amino acids $i$ and $j$ differ respectively by one, two or three nucleotides.

## Figure S3 (supplementary information).

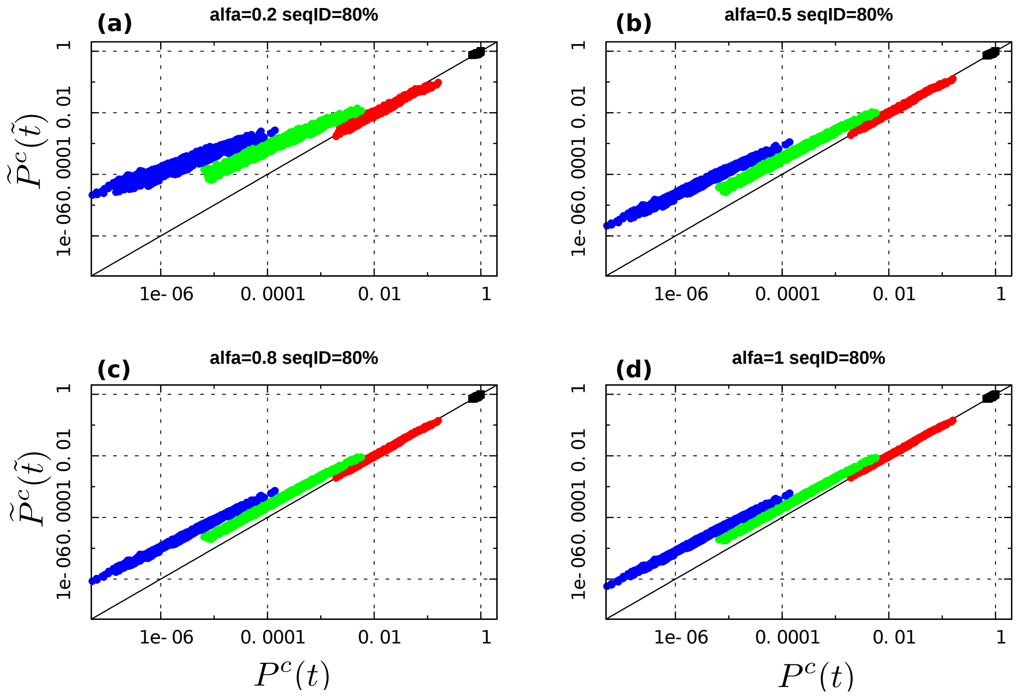


Fig. 3: (a) Entry-by-entry comparison of $P^c(t)$ and $\widetilde{P}^c(\tilde{t})$ in log-log scale, for $t$ and $\tilde{t}$ chosen so that $I^c_M(t) = I^c_{NM}(\tilde{t}) = 0.8$. Each point corresponds to a pair $i, j$ of codons and its $x$-value is given by $P^c_{ij}(t)$, while its $y$-value is $\widetilde{P}^c_{ij}(t)$ where parameter $\alpha$ in the $\gamma$-distribution is set to $\alpha = 0.2$. The black squares are the entries with $i = j$, while red, green and blue points are respectively the entries where codon $i$ and codon $j$ differ by one, two or three nucleotides. Solid line: line $y = x$. (b) Same comparison as in panel (a), but for $\alpha = 0.5$. (c) Same comparison as in panel (a), but for $\alpha = 0.8$. (d) Same comparison as in panel (a), but for $\alpha = 1$.
